# Why should health be a central argument in climate negotiations? Can a MOOC help to bring the message across?

**DOI:** 10.1186/s40985-016-0030-7

**Published:** 2016-10-19

**Authors:** Rainer Sauerborn

**Affiliations:** 1 Institute of Public Health, Heidelberg University, Heidelberg, Germany; 2 Centre de Santé Publique Virchow-Villermé, Université Paris Sorbonne-Cité, Paris, France

**Keywords:** Climate change, Human health, Policy, Messaging, Decision-making

## Abstract

There are four key messages from health for climate negotiations. Two positive ones include (i) health as a motivator for action and policy and (ii) huge health co-benefits to be included in the cost-benefit trade-offs of climate negotiations. Two warning messages: (iii) there are health-based absolute limits of adaptations and (iv) hotter average temperatures will cut work productivity of farmers and other outdoor workers as well as workers in non-air conditioned factories in poor countries. This paper will examine how massive open online courses (MOOCs) have been used in the run-up to this COP to disseminate these four messages to the audience of high-level policy-makers. This required a departure from the classic MOOC format in several ways: duration, focus on decision-making rationale, policy-relevant messages presented in big brush, leaving “traceable accounts” to evidence in two layers of resources provided: essential and “deep dive”.

## Background

This presentation is predicated on the argument that health is more than just one of many other sectors, like agriculture and forestry. Rather, health is an argument, a leverage in otherwise conflict, conflictual and interest-guided debates to promote and motivate climate policy and climate-conscious behaviours [1].

This paper has two parts: First, we consider the four key messages from health for climate policy-makers. Second, we identify challenges in the scientific evidence from health for climate policy. In which areas can we improve to strengthen the evidence base for these health messages for climate policy?

## Main text

There are four such key messages, two of them are positive and two are negative or warnings.

The first positive message is that health is a motivator for citizens’ behaviour change and climate policy-makers. In fact, it is the driving force, why most people care about climate change. So, it is an argument that drives change, which generates energy to do something about climate change.

The second positive message lies in the huge health co-benefits that accrue from climate policies [2, 3]. The somewhat simplifying motto is “what’s good for the climate is good for health” true for many policies, such as enhancing personal mobility or eating less red meat. A huge co-benefit has only recently been identified [4]: black carbon is a climate-active pollutant, which is produced mainly by indoor cooking with biomass, a practice, which is wide-spread particularly in low-income countries. Indoor air-pollution kills about four million people each year, most of them are women and children [5]. So, by doing something for the climate (reducing black carbon), we also do something for our health. Cognitive psychology tells us that positive arguments are much more powerful in inducing change in behaviour and for that matter in policies than the quite negative scenarios that are often put forward. The message is, a climate-friendly planet is a healthier planet.

This said, there are also two negative messages from public health which we see as warning signs or guard-rails for climate policy-makers.

The first of them states that there are absolute health-based limits of adaptation to a world with unfettered climate change, beyond 2° warming. This is based on physiological evidence, i.e. from the way our body works. This is true certainly for our limits of adapting to a warmer world, to higher temperatures, but it also applies to the ability to cope with other diseases that will be increased due to climate change, such as infectious diseases, heart and lung diseases as well as mental diseases [3, 4].

The second negative argument is a health-based economic argument. We know that as temperature rises, our capacity to work is decreasing, so is our work output [6]. This is very bad news for high-temperature tropical and subtropical countries, for which economic growth is a key precondition for development. Not only the outdoor workers, such as construction workers, farmers or traffic policemen are concerned but also the increasing number of factory workers. To date, only a tiny fraction of factories in low- and middle-income countries are air-conditioned. Hence, temperatures inside the factory are likely to be even higher than outside. This argument from heat physiology has a bearing on not only on health effects but also on work productivity.

In the second part of this presentation, we present data indicating research needs to further corroborate these health messages for climate policy. Does the world produce enough health research for policy-makers in the right areas?

There is a good and bad news. The good news is the upward trend of scientific publications, in which health aspects of climate change are studied from 1990 till today. Simultaneously, but with a delay of several years, the Intergovernmental Panel on Climate Change (IPCC) increasingly deals with health aspects in its assessment reports. For example, the term “health” is mentioned 10 times in the first IPCC report in 1990 but more than 2500 times in the last report in 2014 [7]. Interestingly, only 26 % of these references of the world health originated from the health chapter. In the majority of instances, health was mentioned in the chapters of other sectors, such as agriculture, tourism or forestry. One could arguably say that health has become a mainstream concern.

However, when looking at the absolute numbers of publications dealing with health aspects of climate change and comparing them to climate-related publications from other sectors, health research is still significantly behind [7].

The second not so good news is the mismatch between, on the one hand, the topics of published studies and, on the other hand, the topics and evidence which policy-makers want. The latter include studies on the costs and effectiveness of adaptation measures for example. Furthermore, the topics that interest researchers are often not in sync with the imputed size of climate-related health problems. For example, research on the effect of heat waves abound, from Göteborg to Italy, but research on climate impact on malnutrition is very scanty [8], although malnutrition is considered to be one of the main health impacts [3, 4, 9]. It is very telling to compare the research output on different risk factors: the scientific output was about 40,000 papers in between 2002 and 2012. In the same time span, only 300 articles or less than 1 % were published on climate change as a risk factor for health [10].

Beyond the mismatch of health topics studied and those most relevant, there is huge North-South gap in publications. Both as far as authors and as far as the socio-economic study context are concerned, the North dominates the scientific production. This too, needs to see rigorous funding policies to fill the gap [7].

Looking at the policy relationship between a health topic and climate change, we classified research into the following categories (i) impact, (ii) co-benefits, (iii) adaptations, (iv) cost estimates of policies and (v) long-term projections linking health data to climate models. We observed that most health-related research on climate change is on impacts and only recently on co-benefits. Very little research is dedicated to adaptation policies, their costs and effectiveness. Long-term model projections of health impacts are the least frequent subjects of research.

## Conclusions

To sum up, there are four important messages from health for climate policy. These should be undergirded with more research, which focuses better on the evidence needs of policy makers: projection of health impacts in understudied areas, such as malnutrition and health adaptation strategies and their costs are the two major areas that need further research.

## Abbreviations

IPCC: Intergovernmental Panel on Climate Change
